# Photocurrent Spectroscopy of Dark Magnetic Excitons in 2D Multiferroic NiI_2_

**DOI:** 10.1002/advs.202407862

**Published:** 2024-08-09

**Authors:** Dmitry Lebedev, J. Tyler Gish, Ethan S. Garvey, Thomas W. Song, Qunfei Zhou, Luqing Wang, Kenji Watanabe, Takashi Taniguchi, Maria K. Chan, Pierre Darancet, Nathaniel P. Stern, Vinod K. Sangwan, Mark C. Hersam

**Affiliations:** ^1^ Department of Materials Science and Engineering Northwestern University Evanston IL 60208 USA; ^2^ Department of Physics and Astronomy Northwestern University Evanston IL 60208 USA; ^3^ Center for Nanoscale Materials Argonne National Laboratory 9700 South Cass Avenue Lemont IL 60439 USA; ^4^ Northwestern‐Argonne Institute of Science and Engineering 2205 Tech Drive Evanston IL 60208 USA; ^5^ Research Center for Functional Materials National Institute for Materials Science 1‐1 Namiki Tsukuba 305‐0044 Japan; ^6^ International Center for Materials Nanoarchitectonics National Institute for Materials Science 1‐1 Namiki Tsukuba 305‐0044 Japan; ^7^ Department of Chemistry Northwestern University Evanston IL 60208 USA; ^8^ Department of Electrical and Computer Engineering Northwestern University Evanston IL 60208 USA

**Keywords:** 2D materials, antiferromagnetism, dark exciton, multiferroicity, photocurrent

## Abstract

Two‐dimensional (2D) antiferromagnetic (AFM) semiconductors are promising components of opto‐spintronic devices due to terahertz operation frequencies and minimal interactions with stray fields. However, the lack of net magnetization significantly limits the number of experimental techniques available to study the relationship between magnetic order and semiconducting properties. Here, they demonstrate conditions under which photocurrent spectroscopy can be employed to study many‐body magnetic excitons in the 2D AFM semiconductor NiI_2_. The use of photocurrent spectroscopy enables the detection of optically dark magnetic excitons down to bilayer thickness, revealing a high degree of linear polarization that is coupled to the underlying helical AFM order of NiI_2_. In addition to probing the coupling between magnetic order and dark excitons, this work provides strong evidence for the multiferroicity of NiI_2_ down to bilayer thickness, thus demonstrating the utility of photocurrent spectroscopy for revealing subtle opto‐spintronic phenomena in the atomically thin limit.

## Graphical abstract

2D antiferromagnetic (AFM) semiconductors are promising components of opto‐spintronic devices due to terahertz operation frequencies and minimal interactions with stray fields. Here, photocurrent spectroscopy is employed to study the 2D AFM semiconductor NiI_2_, which enables the detection of optically dark magnetic excitons down to bilayer thickness, revealing linear polarization that is coupled to the underlying helical AFM order of NiI_2_.

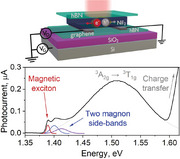

## 1 Introduction

The coupling between magnetic order and optically excited states, such as excitons, has become a central topic of research for van der Waals two‐dimensional (2D) magnets.^[^ ^1^ ^]^ This coupling allows magnetic properties to be probed by studying excitons, which provides opportunities beyond traditional methods such as magnetometry or diffraction‐based tools. E.g., photoluminescence spectroscopy of NiPS_3_ has revealed ultranarrow emission (linewidths less than 0.4 meV) with a high degree of linear polarization.^[^ ^2^ ^]^ This emission is associated with coherent many‐body excitons that are entangled with antiferromagnetic (AFM) order.^[^ ^2a^ ^]^ In this manner, photoluminescence measurements have allowed investigation of the magnetic properties of NiPS_3_, such as the orientation of the Néel vector and critical exponents that characterize the spin dimensionality class.^[^ ^2b^ ^]^ In addition, the interplay between excitons and magnetic order allows for magnetic field control over excitonic emission. For the aforementioned NiPS_3_, the linear polarization angle of photoluminescence has been controlled through the application of an external magnetic field.^[^ ^3^ ^]^ Another 2D magnetically ordered semiconductor, CrSBr, has exhibited strongly coherent exciton‐magnon coupling, which can be tuned by the application of strain or an external magnetic field.^[^ ^4^ ^]^

**Figure 1:**
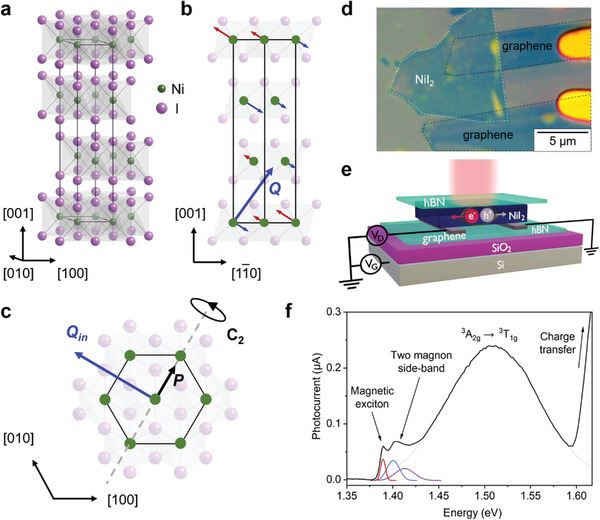
Crystal and magnetic structures, device architecture, and photocurrent spectroscopy of NiI_2_. a) Crystal structure of NiI_2_. The schematic of the ground state helical magnetic order, viewed along [110] direction (b) and [001] direction (c). The spins are perpendicular to the propagation vector Q (0.138a*,0,1.457c*). The view along [001] shows in‐plane component of Q and the direction of electric polarization P along the two‐fold rotation axis. d) Optical micrograph of a FET device fabricated from a bulk NiI_2_ flake. e) Schematic of the photocurrent spectroscopy measurement on the NiI_2_ FET device. f) Photocurrent spectrum of bulk NiI_2_ (black curve) recorded at 7 K, which shows sub‐bandgap transitions, including the magnetic exciton.

Despite these demonstrations, studies exploring the coupling of excitons with magnetic order in the atomically thin limit remain scarce, primarily due to the limited number of 2D magnetically ordered semiconductors, most of which order antiferromagnetically.^[^ ^1b,c^ ^]^ While AFM materials are more robust against external parasitic magnetic fields and enable higher operating frequencies compared to ferromagnetic (FM) materials, their lack of net magnetization limits options for detecting and controlling AFM order.^[^ ^1a,d^ ^]^ NiI_2_, a recently discovered gate‐tunable 2D van der Waals magnetic semiconductor,^[^ ^5^ ^]^ has a helical AFM ground state below the second Néel temperature T_N2_ ≈ 59 K (T_N1_ ≈ 76 K). The helix has a propagation vector **Q** (0.138 a*,0,1.457 c*) such that the spins make an angle of ≈55° with the [001] direction (**Figure** **1**a–c).^[^ ^6^ ^]^ The emerging helical magnetic order at the T_N2_ transition breaks inversion symmetry and drives in‐plane ferroelectricity due to strong Dzyaloshinskii‐Moriya interactions, making NiI_2_ a multiferroic material (Figure 1c).^[^ ^7^ ^]^ Although early reports suggested that this multiferroicity persists down to monolayer thickness,^[^ ^7a^ ^]^ recent studies have concluded that multiferroicity may only persist down to bilayer thickness.^[^ ^8^ ^]^

Recent absorption spectroscopy and resonant inelastic X‐ray scattering (RIXS) studies detected an excitonic peak in bulk NiI_2_ crystals at an energy of 1.384 eV with a linewidth as narrow as 5 meV.^[^ ^9^ ^]^ This peak showed a small degree of linear polarization (<0.2) and by optical absorption measurements disappeared above T_N2_, which suggests that it is coupled to the underlying magnetic order of NiI_2_. However, unlike NiPS_3_, magnetic excitons in NiI_2_ are dark with no observable photoluminescence, which has hindered studies of these magnetic excitons in few‐layer 2D NiI_2_ samples. Moreover, domains and defects (e.g., stacking faults) likely result in the emergence of multiple spin helix propagation directions coexisting within a bulk crystal, thus further complicating the study of correlations between the polarization of excitons and magnetic order NiI_2_.

Here, we employ photocurrent spectroscopy to study the dark quantum‐entangled excitons in the 2D AFM semiconductor NiI_2_ down to bilayer thickness. Although photocurrent spectroscopy has been previously used to study excited states in 2D semiconductors ^[^ ^10^ ^]^ and light helicity detectors have been realized using tunneling photocurrent measurements of 2D magnetically ordered CrI_3_,^[^ ^11^ ^]^ photocurrent spectroscopy has not yet been applied to 2D AFM semiconductors due to limited electrical conductivity at the cryogenic temperatures where magnetic order is established. By leveraging recent advances in the processing of NiI_2_,^[^ ^5^ ^]^ we demonstrate that photocurrent spectroscopy can be performed on 2D NiI_2_ field‐effect transistors (FETs). Contrary to the previous report on bulk crystals,^[^ ^9^ ^]^ we reveal a high degree of linear polarization for magnetic excitons in ultrathin NiI_2_ and show that the polarization direction is correlated with the helical AFM propagation vector. Measurements of few‐layer samples further show that magnetic excitons in NiI_2_ persist down to bilayer thickness with a blue‐shifted exciton energy by 18 meV compared to bulk NiI_2_.

## 2 Results

In our experiments, NiI_2_ was micromechanically exfoliated using Scotch tape, and FETs were assembled by combining polymer‐assisted flake transfer and lithography methods (see Experimental Section and Figure 1d,e). When processed in a manner that avoids chemical degradation, graphene‐contacted NiI_2_ FETs show ambipolar transport at room temperature with electron (hole) field‐effect mobilities of ≈1 cm^2^ V^−1^ s^−1^ (0.01 cm^2^ V^−1^ s^−1^).^[^ ^5^ ^]^ After confirming high‐quality charge transport characteristics, we acquired photocurrent spectra of bulk NiI_2_ by measuring the FET source‐drain current under optical irradiation over an excitation wavelength range of 500–1000 nm (≈1.2–2.5 eV). At room temperature, the photocurrent is observed to increase with photon energy above 1.38 eV (Figure S1, Supporting Information). By assuming an indirect bandgap for bulk NiI_2_ and fitting the linear region of the Tauc plot of the photocurrent spectrum,^[^ ^12^ ^]^ an energy bandgap of 1.40 eV was extracted (Figure S1, Supporting Information), which agrees well with values obtained by absorption and photocurrent measurements of bulk NiI_2_ crystals.^[^ ^13^ ^]^

Low‐temperature photocurrent spectroscopy measurements reveal an increase in the energy bandgap up to 1.577 eV for bulk NiI_2_ at 7 K, as extracted from the photocurrent Tauc plot (Figure 1f; Figure S1, Supporting Information). In addition, four sub‐bandgap peaks are observed at 1.389, 1.400, 1.413, and 1.510 eV (Figure 1f; Figure S2, Supporting Information). Based on absorption measurements of bulk NiI_2_ crystals, these peaks can be assigned to the magnetic exciton (1.389 eV), two‐magnon sideband absorption (1.40–1.41 eV), and the ^3^A_2g_ → ^3^T_1g_ transition (1.510 eV).^[^ ^9^, ^14^ ^]^ Our photocurrent data yield the best fits when the two sidebands are included (Figure S2, Supporting Information), similar to the magnetic exciton in NiPS_3_ that is also accompanied by several sideband peaks.^[^ ^2a^ ^]^ The magnetic exciton at 1.389 eV is of particular interest due to its narrow linewidth (≈6 meV) and entanglement with the underlying AFM order in NiI_2_.^[^ ^2^, ^9^ ^]^ While magnetic excitons can be detected in NiPS_3_ via photoluminescence spectroscopy,^[^ ^2^ ^]^ the magnetic excitons in NiI_2_ are dark and thus have only been observed using absorption spectroscopy and RIXS in bulk crystals in previous work.^[^ ^9^ ^]^ Therefore, photocurrent spectroscopy measurements offer a unique opportunity to probe the magnetic excitons in exfoliated 2D NiI_2_ flakes.

**Figure 2:**
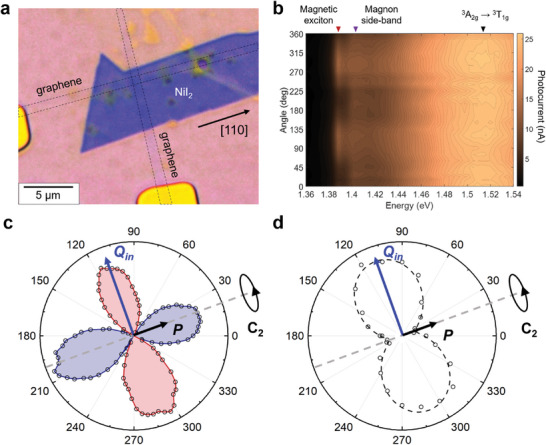
Polarization‐dependent photocurrent characterization. a) An optical micrograph of a vertical FET device fabricated from a bulk NiI_2_ flake. b) Photocurrent as a function of energy and polarization angle of the excitation laser. Optical transitions are marked with triangles; only the magnetic exciton peak shows a polarization‐dependent photocurrent response. c) Linear dichroism data for the bulk NiI_2_ flake shown in (a). Positive lobes (blue color) indicate the direction of the C_2_ rotation axis and electric polarization. d) Polar plot of the magnetic exciton photocurrent extracted from (b); dashed line is the sinusoidal fit. The direction of linear polarization lies along the *Q_in_* component of the magnetic modulation vector, which is orthogonal to the electric polarization.

Photocurrent spectroscopy allows the polarization of the magnetic exciton in NiI_2_ to be measured by irradiating with linearly polarized light. For this experiment, we employed vertical FETs to ensure that the current passes through a single domain of NiI_2_. The vertical FET consists of a NiI_2_ flake sandwiched between two monolayer graphene strips (**Figure** **2**a). Photocurrent measurements reveal that the two‐magnon sidebands and ^3^A_2g_ → ^3^T_1g_ transitions are independent of the polarization of the incident light. However, the magnetic exciton peak shows significant anisotropy with twofold symmetry (Figure 2b; Figure S3, Supporting Information). Fitting the data with a sinusoidal function and calculating the degree of linear polarization as:

(1) $$\rho \;=\frac{I_{⊥}-I_{∥}}{I_{⊥}+I_{∥}}$$

gives *ρ =* 0.81, suggesting strong linear polarization and thus a highly anisotropic magnetic exciton in NiI_2_. Here, $I_{⊥}\left(I_{∥}\right)$ represents the intensity of photocurrent with vertically (horizontally) polarized linear excitation.

To probe the origin of the linear polarization, we first determined the crystal orientation of the NiI_2_ flake by linear dichroism measurements. As established previously, the electric polarization of NiI_2_ (due to its ferroelectricity below the Néel temperature) is pointing along the [110] direction (Figure 2a) and can be probed by linear dichroism.^[^ ^7^ ^]^ We found that the positive lobes on the linear dichroism polar plot are oriented parallel to the NiI_2_ flake edge, allowing that direction to be assigned to [110] (Figure 2c). Since the helix propagation vector **Q** lies in the (110) plane (Figure 1b), the polarization of the magnetic excitons in NiI_2_ is along its in‐plane component, Q_in_ (Figure 2d).

Next, we studied the influence of external factors, such as magnetic field or electrostatic gating, on the magnetic excitons in NiI_2_. The application of an out‐of‐plane magnetic field up to 2.5 T does not change the position or width of the magnetic exciton peaks (Figure S4, Supporting Information). This insensitivity to magnetic field can be explained by the fact that the metamagnetic transitions in NiI_2_ (spin‐flop or spin‐flip) require application of substantially higher out‐of‐plane magnetic fields (>14 T) that are not accessible in our experimental apparatus.^[^ ^7b^ ^]^ Therefore, the rigid magnetic order of NiI_2_ translates into a highly robust nature of the magnetic excitons. Application of an electric field via the gate electrode revealed that the magnetic exciton decreases in energy at both large positive and large negative gate voltages (i.e., 80 V and –60 V, respectively, through a combined dielectric stack of 300 nm thick SiO_2_ and 20 nm thick bottom hBN), although the change in energy is within 1 meV (Figure S5, Supporting Information). Similar to externally applied magnetic fields, the minimal response to electric fields and charge carrier modulation highlights the robustness of the AFM order and magnetic excitons in NiI_2_.

**Figure 3:**
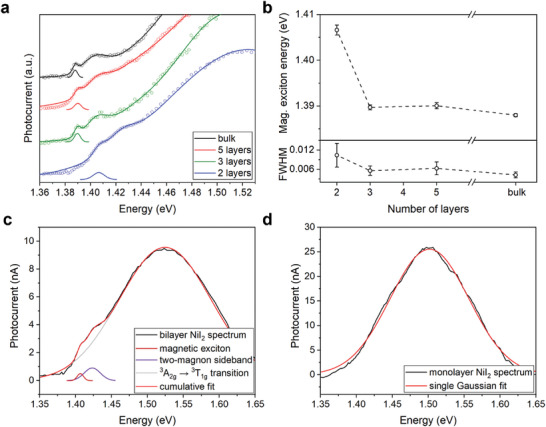
Photocurrent spectroscopy of few‐layer NiI_2_. a) Photocurrent spectra of FET devices fabricated from few‐layer NiI_2_ flakes (open symbols) and their fits (solid lines); the spectra are offset for clarity. The Gaussian peaks represent the magnetic exciton. b) Thickness‐dependent energy of the magnetic exciton in NiI_2_ extracted from the fits of the photocurrent spectra in (a). c,d) Photocurrent spectral fits for bilayer NiI_2_ at 6 K and monolayer NiI_2_ at 2 K, respectively.

Photocurrent spectroscopy was further employed to study the thickness dependence of the spectral response for few‐layer NiI_2_ (**Figure** **3**; Figure S6, Supporting Information). These measurements revealed a negligible shift in magnetic exciton energy down to trilayer thickness. In contrast, the magnetic exciton peak is blue‐shifted by 18 meV in bilayer NiI_2_ (Table S1, Supporting Information). The persistence of the magnetic exciton down to bilayer thickness confirms the multiferroicity of bilayer NiI_2_ in agreement with recent optical studies.^[^ ^8c^ ^]^ On the other hand, the magnetic exciton and magnon peaks were not observed in the photocurrent spectra for monolayer NiI_2_ FETs. Instead, photocurrent measurements on monolayer NiI_2_ only revealed one broad peak, likely corresponding to the ^3^A_2g_ → ^3^T_1g_ transition (Figure 3d).

To understand the shift of the magnetic exciton to higher energies for thin NiI_2_ flakes, we analyzed the photocurrent spectra of NiI_2_ of different thicknesses using Tauc plots (Figure S1, Supporting Information). A slight increase in the bandgap for bilayer and trilayer NiI_2_ by ≈10 meV is observed compared to bulk NiI_2_, which further increases to ≈30 meV for monolayer NiI_2_. These results are consistent with density functional theory modeling of the NiI_2_ bandgap using DFT+U calculations with a classical electrostatic model of the quasiparticle corrections and exciton binding energies that incorporate the heterogeneous dielectric screening effects of hBN.^[^ ^15^ ^]^ Ultimately, this increase in bandgap energy contributes to the increased energy of the magnetic exciton for thinner NiI_2_ flakes.

**Figure 4:**
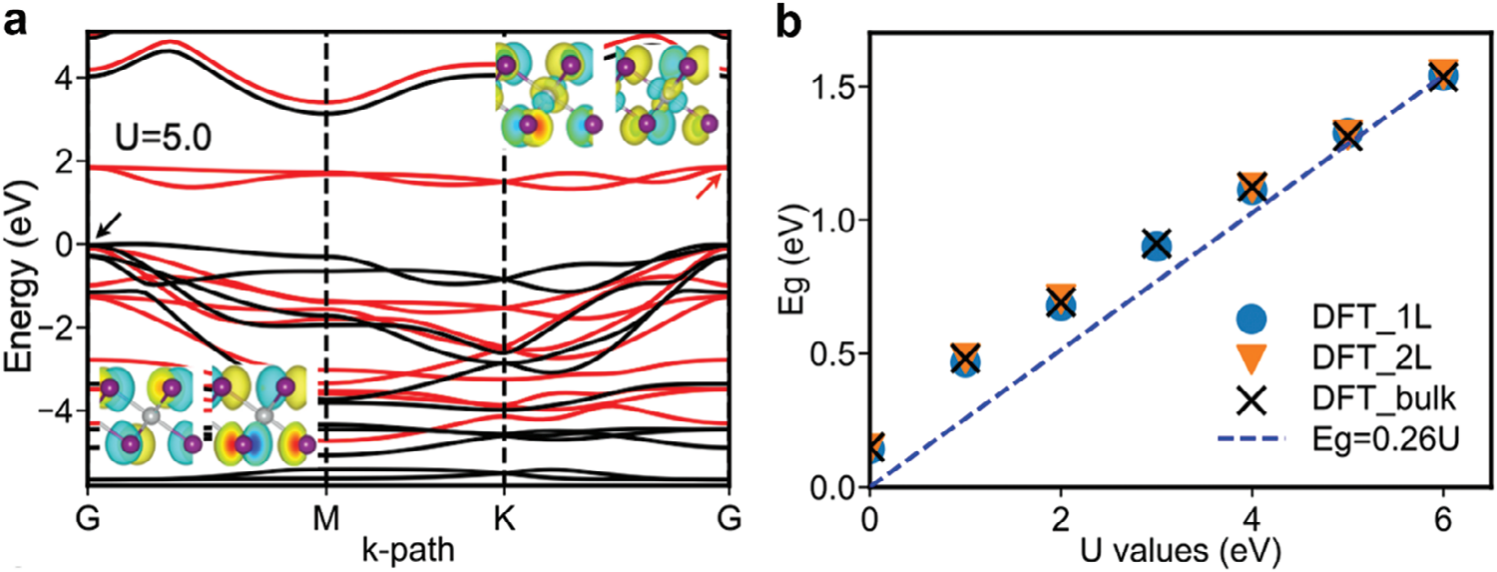
Density functional theory modeling of NiI_2_ band structure. a) Spin‐polarized band structure of monolayer NiI_2_ from DFT+U with U = 5.0 eV. The insets show the wavefunctions for the top valence band and the bottom conduction band at the Γ point, as marked by the black and red arrows, respectively. b) Bandgap of NiI_2_ at different thicknesses as a function of Hubbard U values.

The computed band structure for monolayer NiI_2_ is provided in **Figure** **4** using a Hubbard value U = 5 eV calculated using linear response theory (Figure S7, Supporting Information).^[^ ^16^ ^]^ In order to assess the role of electronic correlations, monolayer NiI_2_ is modeled using an FM configuration. In particular, following the Ni 3d^8^ electronic configuration, the conduction band is comprised of the spin‐down components of the half‐filled e_g_ bands with each Ni atom carrying a magnetic moment of 2𝜇_B_. The valence band has a strong I 5p character, with the half‐filled Ni e_g_ orbitals within 1 eV of the valence band maximum. DFT calculations indicate that the bandgap is correlation‐driven, with the bandgap varying approximately linear with the Hubbard term (Figure 4b). The deviation from a purely linear behavior can be explained by the hybridization of the filled e_g_ orbitals, which are predicted to be the valence band for U < 3 eV with a similar behavior found for bulk NiI_2_ (modeled using AFM configuration; Figures S7 and S8, Supporting Information).

In accordance with the small dispersion of NiI_2_, minimal changes to the bandgap are expected due to quantum confinement. However, the correlation‐driven quasiparticle bandgap and effective values of U are strongly increased as the dielectric screening decreases, with linear response calculations leading to U values of 1.7 eV for bulk NiI_2_ compared to 5 eV for monolayer NiI_2_ in vacuum, leading to a quasiparticle bandgap opening of 0.72 eV (Table S2, Supporting Information; a similar change of 0.87 eV is also predicted by classical electrostatic models ^[^ ^15^ ^]^). The predicted quasiparticle bandgap opening is strongly reduced (0.09 eV) by the presence of the hBN substrate (ε_||_ = 6.9). Importantly, the computed exciton binding energies (Table S2, Supporting Information) are increased by comparable values, resulting in the small increase of the bandgap observed experimentally.

## 3 Discussion

Through the use of cryogenic photocurrent spectroscopy on FET devices, we have studied the magnetic excitons in thick and few‐layer NiI_2_. Previous optical absorption measurements of bulk NiI_2_ crystals showed a minor degree of linear polarization of magnetic excitons (<0.2) and were not able to correlate the polarization with the underlying magnetic order or ferroelectric polarization, likely due to the polycrystalline nature of the samples.^[^ ^9^ ^]^ In contrast, our photocurrent spectroscopy measurements were performed on high‐quality isolated microscopic flakes, which allowed us to reveal the high degree of linear polarization of the magnetic excitons in NiI_2_ (>0.8) and detect the linear polarization orientation with respect to the non‐collinear magnetic order of NiI_2_, namely along the in‐plane component of the helix propagation vector, Q_in_ (Figure 2). These results highlight the power of photocurrent spectroscopy measurements for probing the interplay between optical, electrical, and magnetic properties of 2D magnetic semiconductors. Since the electrical polarization of NiI_2_ pointing along the [110] direction is perpendicular to the linear light polarization that gives the largest exciton photocurrent, we conclude that the ferroelectricity of NiI_2_ does not play a significant role in the properties of the magnetic excitons.

Revealing the high degree of linear polarization of excitons and their correlation to the NiI_2_ magnetic order (i.e., direction of the spin helix propagation vector) has important consequences. First, probing the magnetic order of van der Waals antiferromagnets remains a grand challenge due to the absence of net magnetization. Therefore, development of methods for studying magnetism in atomically thin AFM materials is highly important for the future progress of AFM opto‐spintronics.^[^ ^1b,e^ ^]^ Next, the interplay between magnetic and optoelectronic properties allows the control of excitonic transitions through applied magnetic fields. Indeed, in the case of NiPS_3_, the application of an in‐plane magnetic field results in a spin‐flop transition, which translates into the rotation of the magnetic exciton polarization by 90°.^[^ ^3^ ^]^ Since NiI_2_ has a more complex helical magnetic order than NiPS_3_, its metamagnetic transitions are less explored. We do not see any metamagnetic transitions and hence no change in the energy or width of magnetic exciton peaks with an out‐of‐plane magnetic field up to 2.5 T (Figure S4, Supporting Information). However, the study by Kurumaji et al. on bulk NiI_2_ crystals observed a spin‐flop transition induced by in‐plane magnetic field (starting from 9 T at 50 K), which likely constitutes a rotation of the helical propagation vector by 90° (from Q_in_ ⊥ [110] to Q_in_ ∥ [110]).^[^ ^7b^ ^]^ Further investigations are needed to probe whether such a spin‐flop transition results in the corresponding rotation of polarization direction of the magnetic excitons. Last, the observation of a high degree of linear polarization and correlation to the magnetic order is an important addition to the ongoing studies of the nature of the transition at 1.389 eV. A recent RIXS study of nickel dihalides assigns this transition to a *d‐d* excitation of octahedrally‐coordinated Ni^2+^, suggesting its independence of the presence of long‐range AFM order.^[^ ^17^ ^]^ However, another RIXS study of magnetic excitons in NiPS_3_ revealed exciton‐spin interactions, and found that the propagation of excitons through the AFM lattice is similar to the propagation of a double‐magnon excitation.^[^ ^18^ ^]^ The RIXS studies together with our polarization‐resolved photocurrent spectroscopy measurements call for future experimental and theoretical studies on the nature of the sharp excitonic transitions and their coupling to the underlying long‐range magnetic order in Ni‐based van der Waals AFM semiconductors.

Application of a gate voltage has been shown to be an effective way to change the magnetic coupling between layers and thus magnetism in few‐layer van der Waals magnets such as CrI_3_ and Cr_2_Ge_2_Te_6_.^[^ ^19^ ^]^ However, in the case of NiI_2_, we do not find a strong influence of gate voltage on the magnetic excitons, which highlights the robustness of these transitions with respect to out‐of‐plane electric fields and modulation of the Fermi level. Our probing of the excitons in the lateral FET geometry further allows even stronger electrostatic gating schemes (such as ionic liquids or solid‐state ionic conductors) for modulating the magnetic and optoelectronic properties of NiI_2_.^[^ ^20^ ^]^ Expanding this method beyond a single‐material FET geometry to heterostructure samples also holds promise for studying interfacial effects, proximity coupling, and manipulation of many‐body excitons in the atomically thin limit.

Last, by studying few‐layer NiI_2_ FET devices, we observe the magnetic excitons down to bilayer thickness, which implies that bilayer NiI_2_ has a similar helical magnetic order to bulk NiI_2_. In the case of monolayer NiI_2_, we could only detect the ^3^A_2g_ → ^3^T_1g_ transition, and do not observe the magnetic exciton or magnon sideband peaks (Figure 3). One potential reason for this observation is the broadening of the magnetic exciton and magnon transitions in monolayer NiI_2_ flakes such that their detection is occluded due to the overlap with the neighboring ^3^A_2g_ → ^3^T_1g_ transition. Similar broadening was recently observed for the magnetic exciton in bilayer NiPS_3_, although the exciton in NiPS_3_ is bright and well‐separated from other bands in the spectrum, making it easier to detect via photoluminescence spectroscopy.^[^ ^2b^ ^]^ Another possible explanation is the absence of the magnetic exciton in monolayer NiI_2_, which would point to differences in the long‐range magnetic or ferroelectric order in the monolayer limit for NiI_2_.^[^ ^7^, ^21^ ^]^ This possibility is consistent with recent optical studies that have questioned the originally postulated multiferroicity of monolayer NiI_2_.^[^ ^8^ ^]^

## 4 Conclusions

In conclusion, photocurrent spectroscopy measurements enable electrical probing of quantum many‐body magnetic excitons in the 2D AFM semiconductor NiI_2_. This optically dark magnetic exciton is detected down to bilayer thickness, which is consistent with the helical ground state magnetic order and multiferroicity of bilayer NiI_2_. Photocurrent spectroscopy also reveals a high degree of linear polarization of the magnetic excitons in NiI_2_ as well as coupling to the underlying helical magnetic order. The magnetoelectric nature of NiI_2_ enables potential polarization control using external magnetic and electric fields. For instance, the application of the in‐plane magnetic field is known to transform NiI_2_ into another multiferroic phase, which is proposed to have a spin structure with Q_in_ parallel to [110], as was observed in similar multiferroics MnI_2_ and CoI_2_.^[^ ^7^, ^22^ ^]^ Consequently, this work is likely to be of high interest for emerging efforts to realize and exploit opto‐spintronic and related quantum phenomena in 2D magnetically ordered semiconductors.

## 5 Experimental Section

### NiI_2_ Crystal Growth and Exfoliation

NiI_2_ crystals were grown by the chemical vapor transport method according to previously published procedures, and the quality of the crystals was verified by Raman spectroscopy and charge transport measurements of NiI_2_ FETs.^[^ ^5^, ^6^ ^]^ Few‐layer NiI_2_ flakes were micromechanically exfoliated from the bulk crystal onto 300 nm thick SiO_2_/Si substrates using Scotch tape inside an inert nitrogen atmosphere glovebox. The thickness of the resulting exfoliated NiI_2_ flakes was identified based on optical contrast and variable temperature charge transport measurements described below.^[^ ^5^ ^]^

### Device Fabrication

FET device fabrication was performed using polymer‐assisted flake transfer in an inert nitrogen atmosphere glovebox and previously published lithography methods.^[^ ^5^ ^]^ Device assembly was initiated by picking up the top hexagonal boron nitride (hBN) flake, followed by sequential pick‐up of other flakes and landing the heterostructure on pre‐patterned metal contacts.

### Electrical Transport, Photoconductivity, and Photocurrent Spectroscopy Measurements

Device measurements were performed in Lakeshore CRX 4K and Lakeshore CRX‐VF probe stations using Keithley Source Meter 2 400 units. Photocurrent spectroscopy measurements were performed using a Lakeshore CRX‐VF probe station and a SuperK Extreme EXR‐20 laser (NKT Photonics) by varying the wavelength of the incident beam from 500 to 1000 nm using an LLTF‐VIS monochromator. The laser was focused using a lens down to a spot size of ≈100 µm and global power on the order of 100 µW, translating to an intensity on the order of 1 µW µm⁻^2^ in the center of the spot. Identical photocurrent spectra were obtained using a 50x long working distance objective with a spot size of 4 µm (with the laser spot aligned entirely within the channel of the NiI_2_ FET) at similar intensities. The laser was modulated with a mechanical chopper, and the signal was detected using an SR830 lock‐in amplifier (Stanford Research). The laser was calibrated using an IntelliCal Ne/Ar source (Princeton Instruments) and iHR320 spectrometer equipped with a syn‐plus CCD camera (Horiba Scientific).

### Linear Dichroism Measurements

Linear dichroism measurements were carried out with samples mounted in a closed cycle variable temperature cryostat (Opticool, Quantum Design). The CW 2.33 eV laser was focused onto the sample with a long working distance 50x objective using a homebuilt microscope setup. The laser was modulated with a mechanical chopper before being linearly polarized and sent through a photo‐elastic modulator (PEM). The PEM was set to have a maximum retardance of 0.5λ with a fast axis at 45° with respect to the input polarization. A half‐waveplate was used to rotate the modulated polarization with respect to the crystal axes before being focused onto the sample with a 100x objective. The linear dichroism signal was collected in reflection geometry and directed to a Thorlabs avalanche photodiode for lock‐in detection.

### Density Functional Theory Calculations

Structural optimization and electronic band structures were obtained from density functional theory (DFT) and DFT+U calculations that were performed using the Vienna Ab initio Simulation Package (VASP) ^[^ ^23^ ^]^ with projector augmented wave (PAW) pseudopotentials and Perdew‐Burke‐Ernzerhof (PBE) parameterization of the generalized gradient approximation (GGA) exchange‐correlation functional.^[^ ^23a^ ^]^ For the Brillouin zone integration, a k‐point density of ≈40 Å^−1^ was used. Convergences of the total and electronic energies were 10^−5^ eV/atom and <10^−6^ eV, respectively. A vacuum layer larger than 16 Å was chosen to minimize spurious interactions between the periodic layers. A plane‐wave cutoff energy of 600 eV was used. The van der Waals dispersion energy correction used the Grimme D3 method.^[^ ^24^ ^]^ The Hubbard U term was added to describe the strongly localized Ni 3d electrons. Its values for bulk and 2D NiI_2_ with different thicknesses were determined using a self‐consistent approach ^[^ ^16a^ ^]^ based on a linear‐response method.^[^ ^16b^ ^]^ In order to correct the self‐energy errors in DFT calculations and account for the dielectric screening effects of the hBN substrate, a classical electrostatic image model ^[^ ^15^ ^]^ was used on top of PBE+U calculations for the bandgap of NiI_2_. The dielectric constants for hBN and NiI_2_ are 6.9 ^[^ ^25^ ^]^ and 9.65 (calculated from the random phase approximation), respectively. The exciton binding energies were computed using an effective mass theory of the excitons,^[^ ^15c^ ^]^ with consideration of the dielectric screening effect with and without the hBN substrate. The determination of the image plane position for NiI_2_ is shown in Figure S9 (Supporting Information).

## Conflict of Interest

The authors declare no conflict of interest.

## Author Contributions

D.L. and J.T.G contributed equally to this work. D.L., J.T.G., V.K.S., and M.C.H. devised the principal objectives of the project. D.L. grew the NiI_2_ crystals by the chemical vapor transport method. J.T.G., D.L., and T.W.S. exfoliated the NiI_2_ flakes and fabricated the FET devices. D.L., J.T.G., and T.W.S. performed the photocurrent measurements with the help of V.K.S. E.S.G. performed low‐temperature linear dichroism measurements under the supervision of N.P.S.. K.W. and T.T. provided the hBN crystals. L.W. and Q.Z. performed the DFT calculations under the supervision of M.K.C. and P.D.. M.C.H. supervised the project. D.L., J.T.G., and M.C.H. wrote the manuscript with input from all authors.

## Supporting information

Supporting Information

## Data Availability

The data that support the findings of this study are available from the corresponding author upon reasonable request.
