# Supplementary material for: Photocurrent Spectroscopy of Dark Magnetic Excitons in 2D Multiferroic NiI2
Source: Adv Sci (Weinh). 2024 Aug 9;11(38):2407862. doi: 10.1002/advs.202407862 (PMC11481216; doi:10.1002/advs.202407862)
Supplement: Supplementary file 1 — Supporting Information [file ADVS-11-2407862-s001.pdf]

ADVANCED SCIENCE
Open Access

Supporting Information

for *Adv. Sci.*, DOI 10.1002/advs.202407862

Photocurrent Spectroscopy of Dark Magnetic Excitons in 2D Multiferroic NiI_2

Dmitry Lebedev, J. Tyler Gish, Ethan S. Garvey, Thomas W. Song, Qunfei Zhou, Luqing Wang, Kenji Watanabe, Takashi Taniguchi, Maria K. Chan, Pierre Darancet, Nathaniel P. Stern, Vinod K. Sangwan and Mark C. Hersam**

Supporting Information

Photocurrent spectroscopy of dark magnetic excitons in two-dimensional multiferroic NiI_2

Dmitry Lebedev,[∇,1] *J. Tyler Gish,*[∇,1] *Ethan S. Garvey,*[2] *Thomas W. Song,*[1] *Qunfei Zhou,*[3,4] *Luqing Wang,*[3,4] *Kenji Watanabe,*[5] *Takashi Taniguchi,*[6] *Maria K. Chan,*[3,4] *Pierre Darancet,*[3,4] *Nathaniel P. Stern,*[2] *Vinod K. Sangwan,*[1,*] *and Mark C. Hersam*[1,7,8,*]

1. Department of Materials Science and Engineering, Northwestern University, Evanston, IL 60208 USA
2. Department of Physics and Astronomy, Northwestern University, Evanston, IL 60208 USA
3. Center for Nanoscale Materials, Argonne National Laboratory, 9700 South Cass Avenue, Lemont, IL60439, USA
4. Northwestern-Argonne Institute of Science and Engineering, 2205 Tech Drive, Evanston, IL 60208, USA
5. Research Center for Functional Materials, National Institute for Materials Science, 1-1 Namiki, Tsukuba 305-0044, Japan
6. International Center for Materials Nanoarchitectonics, National Institute for Materials Science, 1-1 Namiki, Tsukuba 305-0044, Japan
7. Department of Chemistry, Northwestern University, Evanston, IL 60208 USA
8. Department of Electrical and Computer Engineering, Northwestern University, Evanston, IL 60208 USA

*e-mail: vinod.sangwan@northwestern.edu, m-hersam@northwestern.edu

∇ These authors contributed equally to this work.

Keywords: 2D materials; antiferromagnetism; multiferroicity; dark exciton; photocurrent

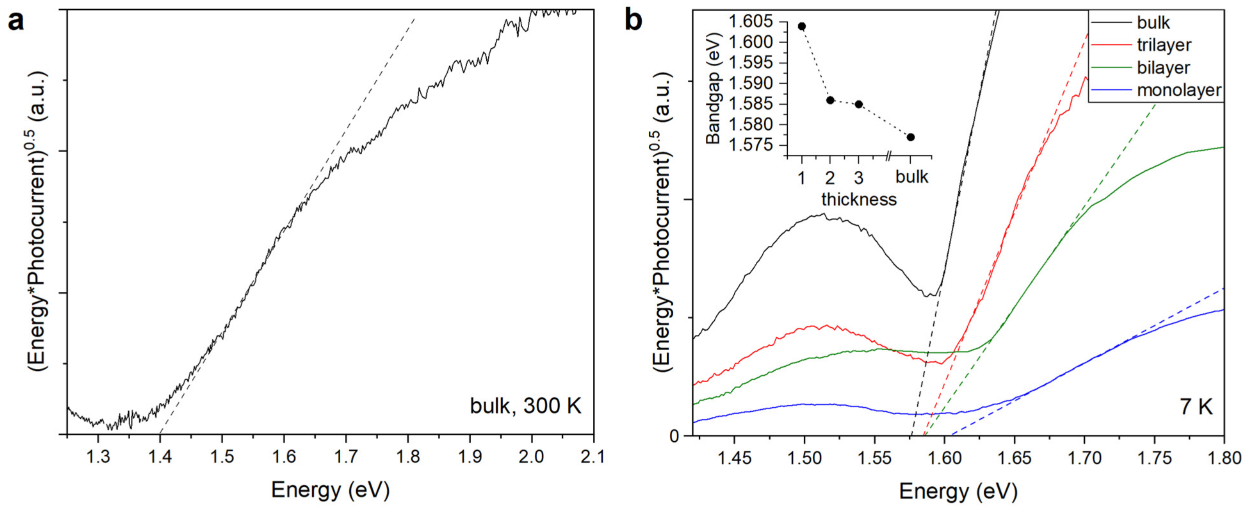

Fig. S1. Optical bandgap of NiI_2. a, Linear fitting of photocurrent spectroscopy Tauc plot of a bulk NiI_2 FET at room temperature. **b,** Linear fitting of photocurrent spectroscopy Tauc plots of NiI_2 FETs of different thickness at 7 K. Inset: Optical gap as a function of NiI_2 thickness at 7 K.

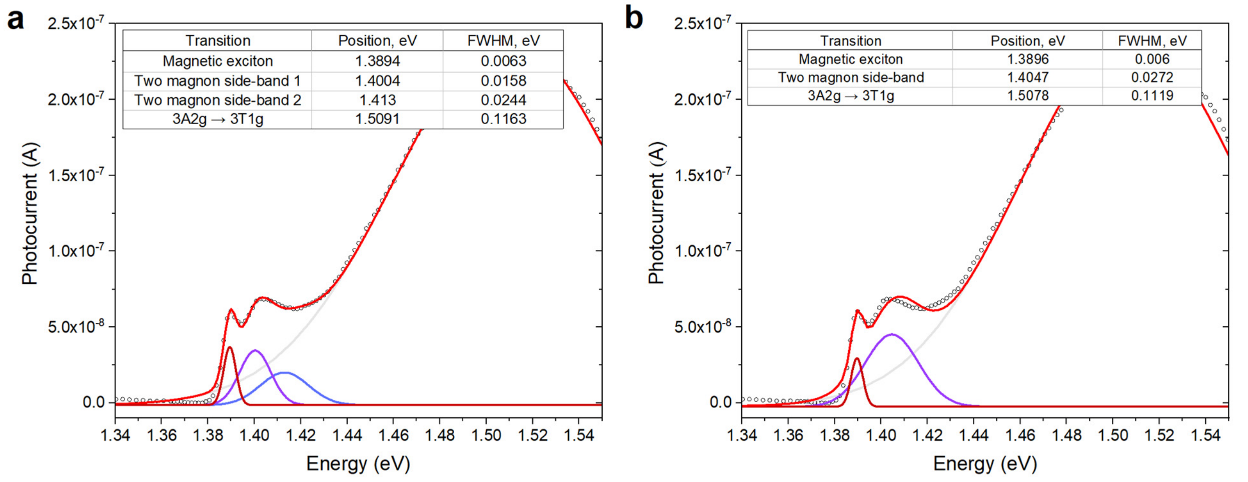

Fig. S2. Sub-bandgap transitions of bulk NiI_2 measured by photocurrent spectroscopy. Fitting the photocurrent spectra of bulk NiI_2 at 7 K with either (**a**) two sidebands or (**b**) one sideband. Higher quality fits are obtained by including multiple sidebands. Open symbols: experimental data, red curve – cumulative fit.

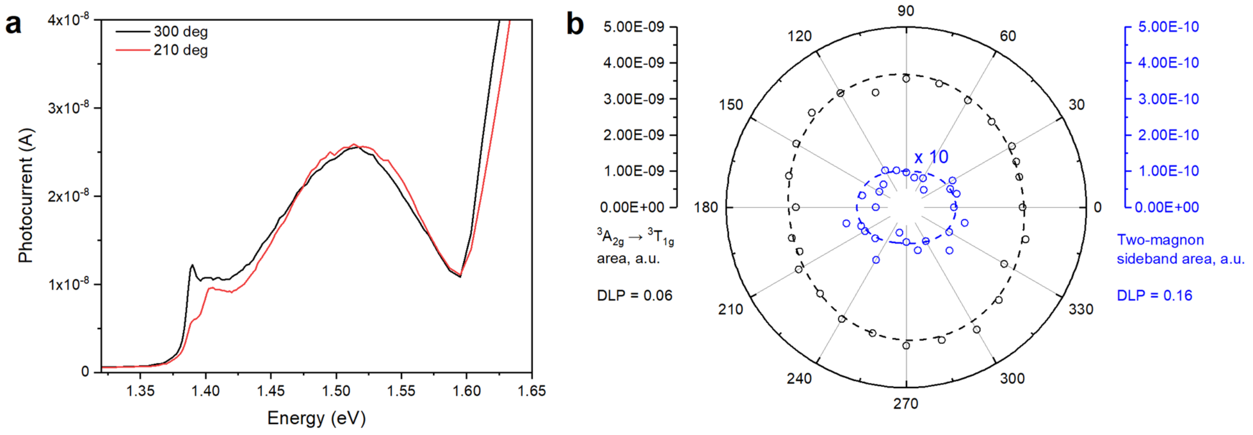

Fig. S3. Photocurrent spectra of NiI_2 with linearly polarized excitation. **a,** Photocurrent spectra of bulk NiI_2 recorded with two orthogonal excitation polarizations at 7 K. **b,** Integrated intensity of the $^3A_{2g} \rightarrow {}^3T_{1g}$ and two-magnon sideband transition as a function of excitation polarization angle. Fitting the data with sinusoidal functions (dashed lines) results in negligible degrees of linear polarization of 0.06 and 0.16, respectively. In order to decrease the number of variables in the fits, the magnon sidebands were fitted as one peak.

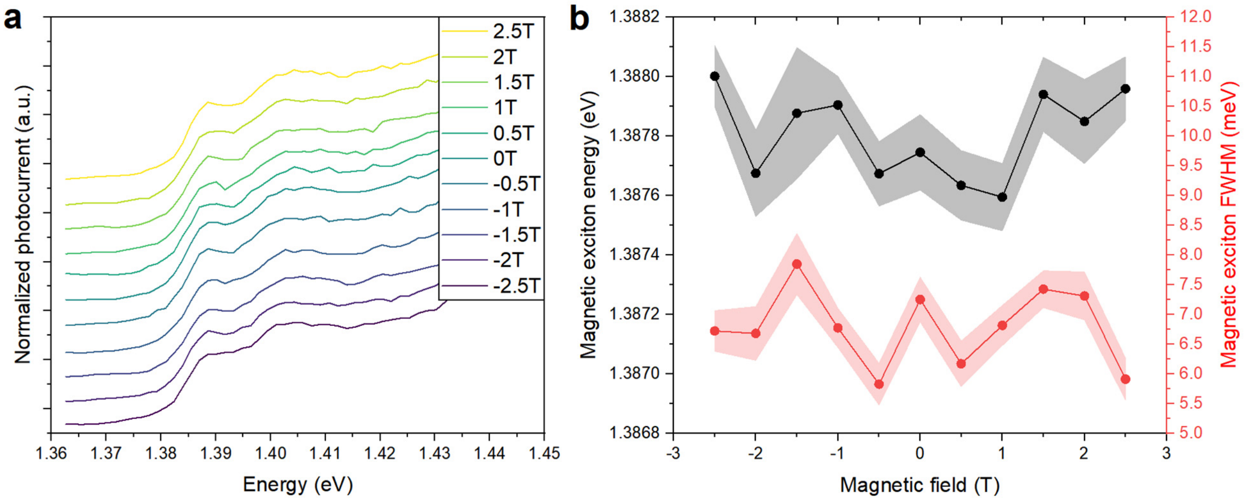

Fig. S4. Magnetic field dependence of the NiI_2 magnetic exciton. a, Photocurrent spectra of bulk NiI_2 recorded at different magnetic fields (out-of-plane) at 12 K using a lateral FET geometry. **b,** Energy and full width at half maximum (FWHM) for the magnetic excitons in NiI_2 as a function of magnetic field, showing no change in energy or width due to the robust nature of the magnetic exciton. Shaded areas represent uncertainties obtained from the fitting.

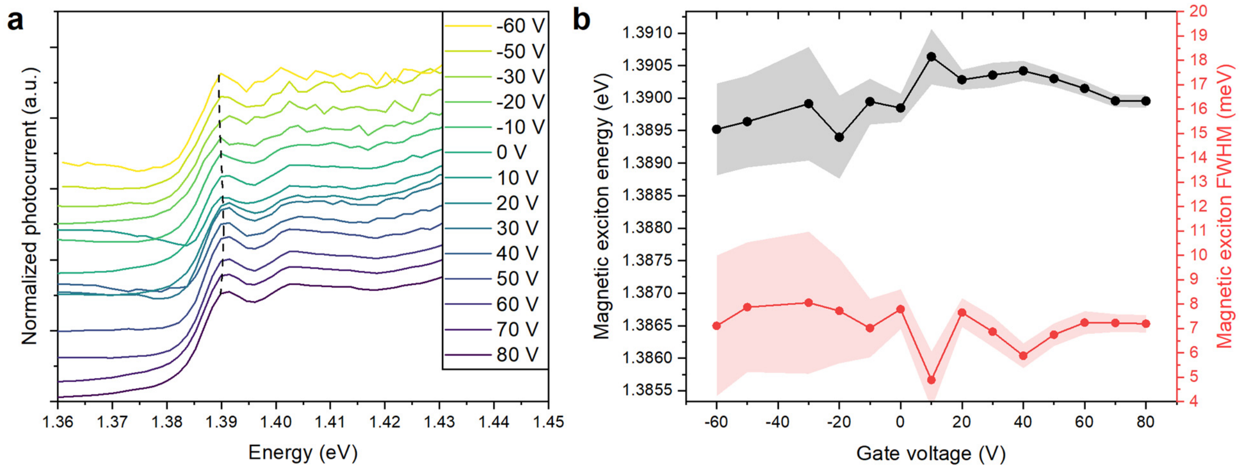

Fig. S5. Gate dependence of the NiI_2 magnetic exciton. a, Photocurrent spectra of bulk NiI_2 recorded at different gate voltages at 7 K using a vertical FET geometry. The dashed line is drawn as a guide for the eye. **b,** Energy and full width at half maximum (FWHM) for the magnetic excitons in NiI_2 as a function of gate voltage. Both strong negative and strong positive gate biases cause a decrease in the magnetic exciton energy. Shaded areas represent uncertainties obtained from the fitting.

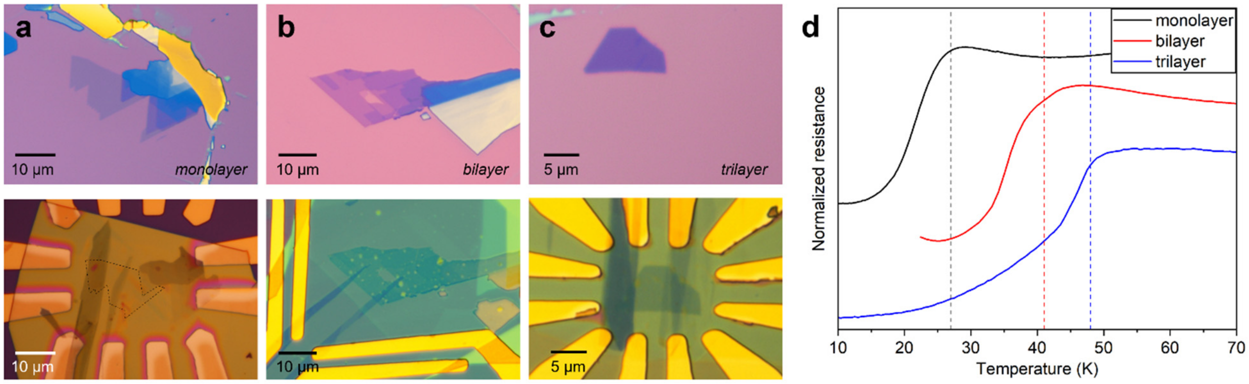

Fig. S6. Optical micrographs of few-layer NiI_2 flakes and corresponding FET devices. a, Monolayer, **b**, bilayer, and **c**, trilayer NiI_2 flakes on a SiO_2/Si substrate (top row) and the corresponding devices (bottom row). **d**, Normalized resistance versus temperature curves (with the vertical offset for clarity) for the devices shown in **a**-**c**. Vertical dashed lines mark the Néel temperature, which decreases with decreasing thickness,[1] corroborating the thickness assignment.

Table S1. Magnetic excitons and two magnon sidebands in bulk and few-layer NiI_2.

Thickness	Magnetic exciton (eV)	FWHM of magnetic exciton (eV)	Two-magnon sideband (eV)	FWHM of two-magnon sideband (eV)
bulk vertical	1.3894	0.0063	1.4004<br>1.4130	0.0158<br>0.0244
bulk lateral	1.3880	0.0043	1.4030<br>1.4264	0.0224<br>0.0457
5 layers lateral	1.3901	0.0063	1.4047<br>1.4258	0.0210<br>0.0391
3 layers lateral	1.3897	0.0056	1.4046<br>1.4282	0.0192<br>0.0493
2 layers lateral	1.4066	0.0104	1.4232	0.0263

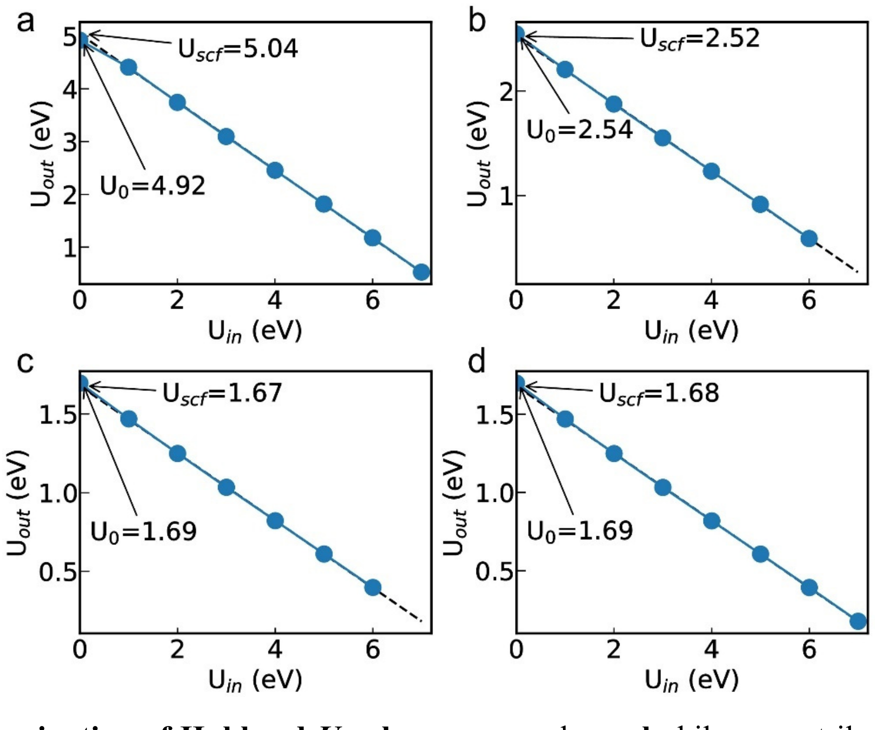

Fig. S7. Determination of Hubbard *U* values. **a,** monolayer, **b,** bilayer, **c,** trilayer and **d,** bulk NiI_2. U_0 are the values from the linear-response approach, and U_{scf} are the results from the self-consistent approach.

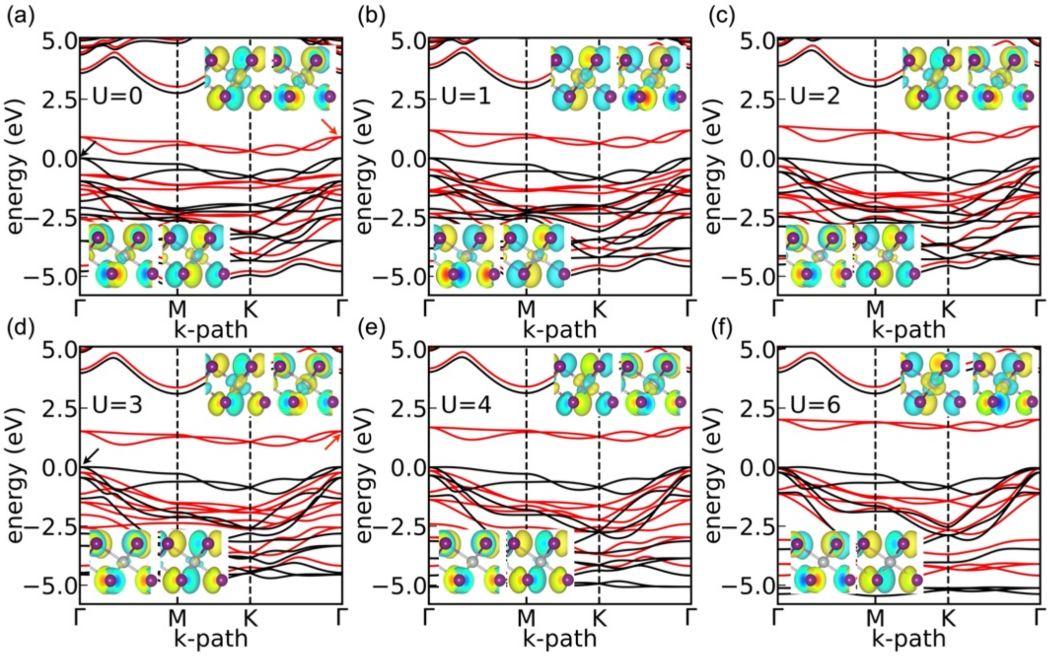

Fig. S8. Spin-polarized electronic band structure of monolayer NiI_2 from DFT+U with different U values. The insets are the same as in Fig. 4a, which shows the decreasing d character to the top-most valence bands with an increase of U.

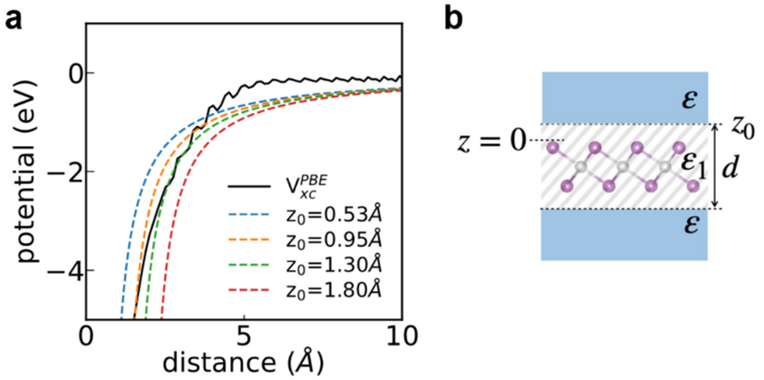

Fig. S9. The image plane position and slab geometry for NiI_2. **a**, z = 1.3 Å, determined when the exchange-correlation potential from PBE and image potential with image plane z_0 having the same tangent point. **b**, The slab geometry for calculation of dielectric screening effects on the bandgap of 2D NiI_2. z_0 is the image plane position. The dielectric constant is ε = 6.9 for hBN[2] and ε_1 = 9.65 for NiI_2 (calculated using DFT). The thicknesses d are included in Table S2.

Table S2. Results for the Hubbard U values determined as shown in Fig. S7. The effective mass of electrons, heavy and light holes, m_e, m_{HH}, m_{LH}, respectively, in units of electron mass; the effective thickness d (in units of Å); the dielectric screening effects on the quasiparticle band gaps for 2D NiI_2 in vacuum and encapsulated with bulk hBN at the top and bottom, $\Sigma(\varepsilon_0)$ and $\Sigma(\varepsilon_{BN})$, respectively, in units of eV; the exciton binding energies for 2D NiI_2 in vacuum and encapsulated with bulk hBN at the top and bottom, $E_b(\varepsilon_0)$ and $E_b(\varepsilon_{BN})$, respectively, in units of eV, which is computed using the effective-mass theory for excitons.[3]

thickness	U (eV)	m_e	m_{HH}	m_{LH}	d	$\Sigma(\varepsilon_0)$	$\Sigma(\varepsilon_{BN})$	$E_b(\varepsilon_0)$	$E_b(\varepsilon_{BN})$
1 layer	5	0.76	1.71	0.27	5.74	0.87	0.09	1.07	0.38
2 layers	2.5	0.76	1.96	0.23	12.48	0.40	0.04	0.70	0.36
3 layers	1.7	0.73	2.52	0.24	19.06	0.26	0.03	0.59	0.36
bulk	1.7	0.42	2.61	0.24	-	-	-	-	-

References

1. Lebedev, D.; Gish, J. T.; Garvey, E. S.; Stanev, T. K.; Choi, J.; Georgopoulos, L.; Song, T. W.; Park, H. Y.; Watanabe, K.; Taniguchi, T.; Stern, N. P.; Sangwan, V. K.; Hersam, M. C., Electrical interrogation of thickness-dependent multiferroic phase transitions in the 2D antiferromagnetic semiconductor NiI_2. *Adv. Funct. Mat.* **2023,** *33* (12), 2212568.

2. Laturia, A.; Van de Put, M. L.; Vandenberghe, W. G., Dielectric properties of hexagonal boron nitride and transition metal dichalcogenides: from monolayer to bulk. *npj 2D Mater. Appl.* **2018,** *2* (1), 6.

3. Cho, Y.; Berkelbach, T. C., Environmentally sensitive theory of electronic and optical transitions in atomically thin semiconductors. *Phys. Rev. B* **2018,** *97* (4), 041409(R).
